# A Rare Case of Systemic Lupus Erythematosus Manifesting as Genital Ulcers

**DOI:** 10.7759/cureus.23818

**Published:** 2022-04-04

**Authors:** Gokul Paidi, Himaja V

**Affiliations:** 1 Family Medicine, The Median Clinic, Grandview, USA; 2 Obstetrics and Gynecology, SSIMS & RC, Davangere, IND

**Keywords:** systemic lupus erythematosus, chrons disease, genital ulcers, vasculitis, autoimmune

## Abstract

Systemic lupus erythematosus (SLE) is a chronic autoimmune disease affecting multiple organ systems. In this report, we discuss the case of a patient with a history of idiopathic thrombocytopenic purpura (ITP), hypothyroidism, SLE, and Crohn's disease (CD) who presented to the emergency room with fever, burning micturition, abdominal pain, and perineal ulcers. Upon subsequent treatment for urinary tract infections (UTI) and negative evaluations for an infectious cause of genital ulcers like sexually transmitted diseases, the etiology of ulcers was found to be SLE. This case report highlights the importance of including SLE ulcers in the differential diagnosis when an SLE patient presents with genital ulcers and the importance of ruling out an SLE vs. infection or non-infection as the cause of ulcers in a former SLE patient.

## Introduction

Systemic lupus erythematosus (SLE) is an autoimmune disorder commonly seen among young adult females (15-35 years old), where the body’s immune system targets its own healthy organs [1]. SLE affects several organs like the heart, joints, skin, lungs, digestive system, blood vessels, kidneys, and central nervous system. Although manifestations of SLE purely as genital ulcers are rare, ruling out SLE is of paramount importance, especially in a patient with a previous history of SLE. Only a small number of cases of SLE genital ulcers have been published in the literature [2]. Over time, several classifications of cutaneous lesions in lupus erythematosus have been proposed [3]. Two prominent forms associated with SLE are the acute malar and chronic discoid lupus erythematosus (DLE) rash [4]. Cutaneous lupus erythematosus (CLE) is associated with a broad range of dermatologic symptoms. DLE, which is a form of CLE, is characterized by erythematous patches and plaques accompanied by an adherent scale. DLE mostly affects areas exposed to the sun, as well as oral mucosa. Involvement of the genital area is extremely rare [5]. We describe the clinical course of a young female patient with a history of idiopathic thrombocytopenic purpura (ITP), Crohn’s disease (CD), SLE, and hypothyroidism who presented with a recurrent history of chronic diarrhea, perianal ulcers, the passage of blood in stools, and anal pain.

## Case presentation

**Figure 1:**
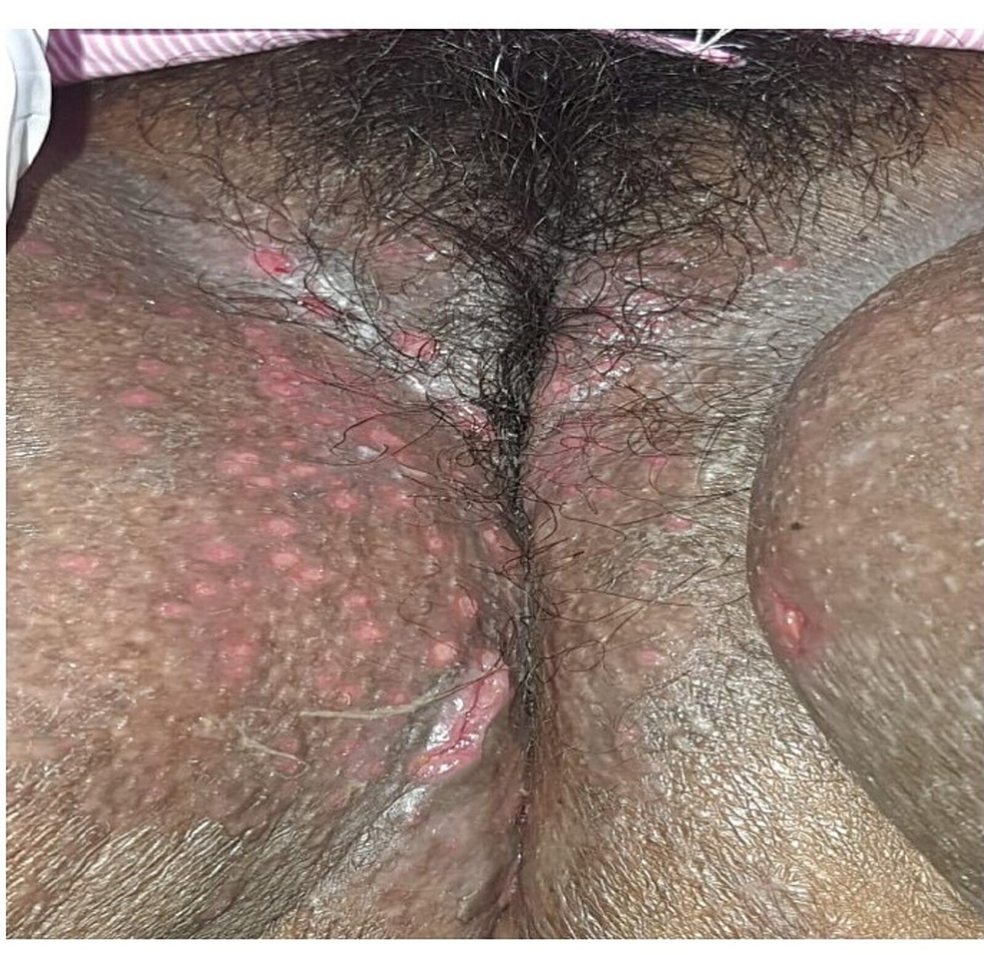
Photographic image showing ulcers on both sides of the labia majora

**Figure 2:**
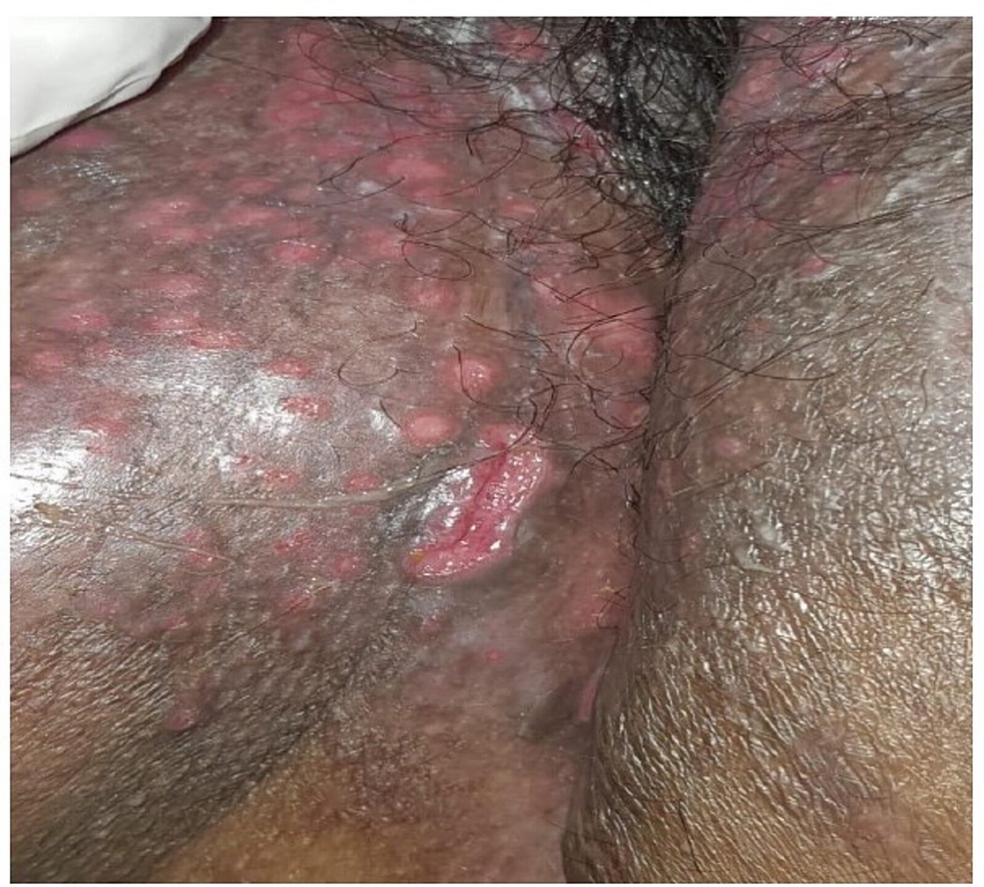
Photographic image showing a clear and closer view of the ulcers

A 28-year-old female with a known medical history of ITP, CD, SLE, and hypothyroidism was admitted due to fever and severe burning micturition for two weeks, which had been exacerbated in the past three to four days along with the appearance of body aches, loose stools, vomiting, and perineal discomfort after passing stool. The patient also had ulcers in the perineum. The ulcers were tender with bilateral multiple papular lesions without lymphadenopathy (Figures 1, 2). The pelvic examination was painful and there was no vaginal discharge. The ulcers in the genital area were initially suspected to be a fungal infection, superimposed bacterial infection, or vulvar CD.

**Figure 3:**
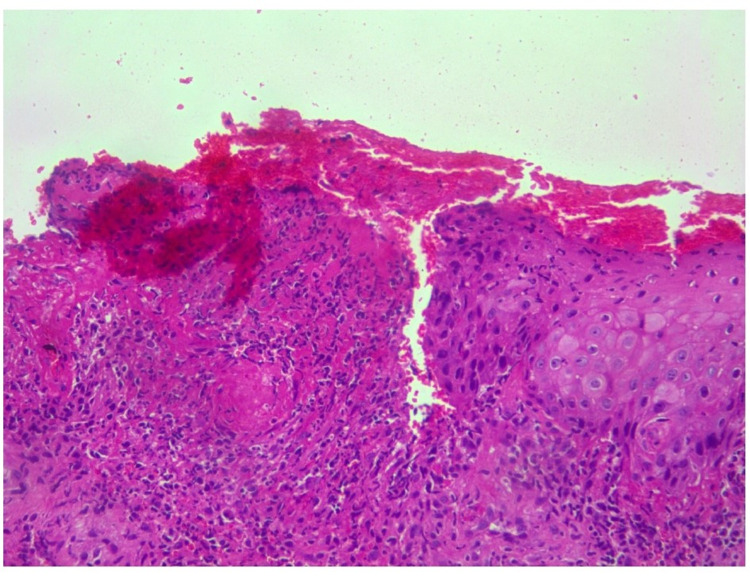
Ulcerated epidermis with dermis showing dense inflammatory cells and fibrinoid necrosis of vessel wall

**Figure 4:**
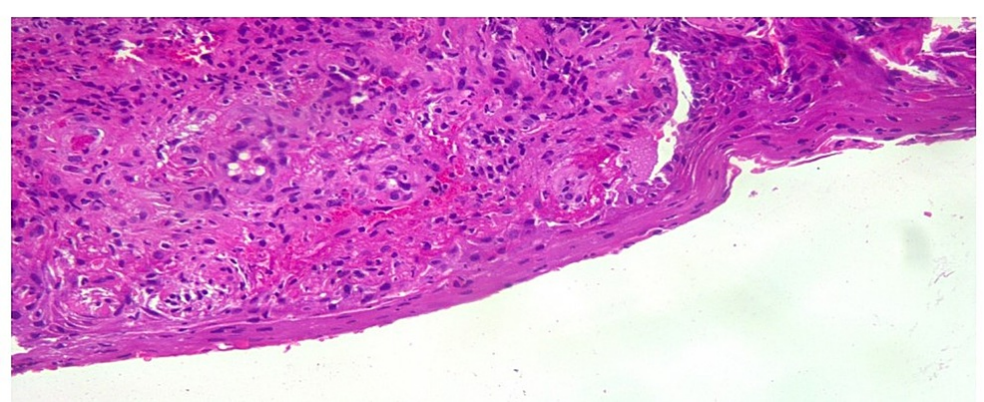
Thinned-out epidermis with thick-walled vessels

**Figure 5:**
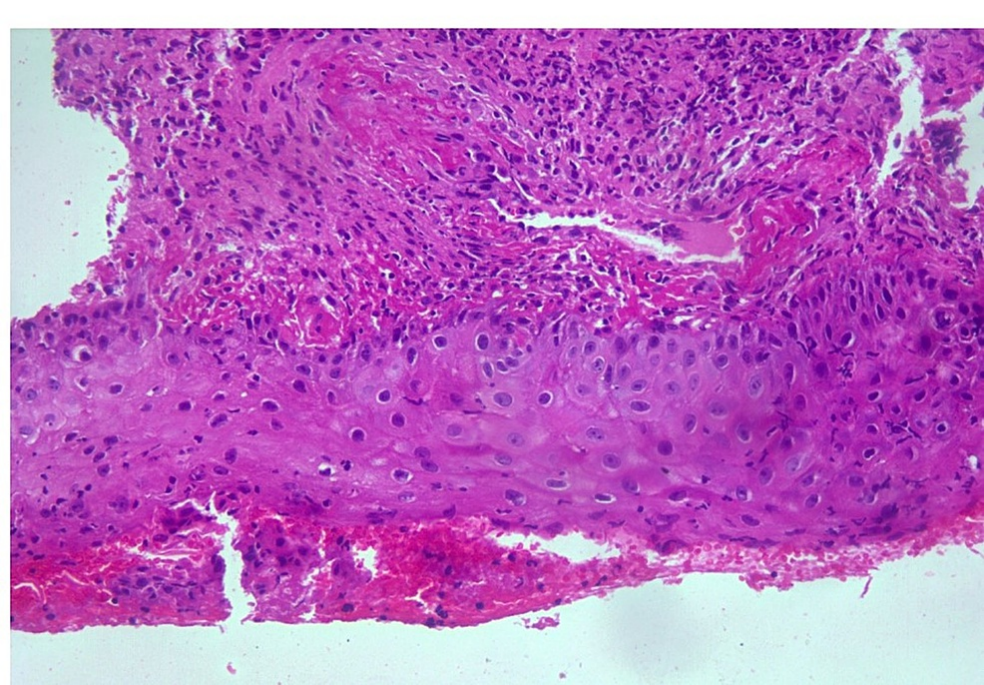
Fibrinoid necrosis of vessel wall with mural thrombi in the dermis

The patient was admitted and started on fluids; blood and metabolic profile, blood and urine cultures along with coronavirus disease 2019 (COVID-19) test, and CT of abdomen and pelvis were ordered. Blood cultures showed no growth and urine cultures showed Klebsiella pneumoniae. The complete metabolic panel (CMP) was normal. CT of the abdomen and pelvis showed no abnormality. The patient was started on electrolyte fluids. She was diagnosed with Klebsiella pneumonia-associated urinary tract infection (UTI) and started on antibiotics. A genital examination revealed multiple ulcers on the perineum and suspected fungal infection with a secondary bacterial infection. The vulvar CD was also suspected and she was started on hydrocortisone. Vulvar biopsy and fungal swab test were ordered. The fungal swab test was negative. A wedge biopsy of genital ulcer tissue bit measuring 0.8 x 0.6 cm was done. Histopathological examination of the biopsy specimen showed epidermis with irregular acanthosis with ulceration and dermis infiltrated by dense lymphoplasmacytic infiltrates, with few neutrophils. The vessel wall showed fibrinoid necrosis of the vessel wall, and a few vessels showed mural thrombus and thick-walled vessels (Figures 3-5). No epithelioid granulomas were seen. Impression features suggested lupus erythematosus. Although immunofluorescence studies were suggested, they could not be performed due to cost constraints. Her condition subsequently improved with intravenous hydrocortisone 20 mg twice a day and ulcers started healing. She was eventually discharged after achieving hemodynamic stabilization.

## Discussion

SLE usually involves multiple systems and its presentations can be varying. Cutaneous manifestations include malar rash, discoid rash, photosensitivity, mucosal rash, lupus panniculitis, lichenoid small vessel cutaneous leukocytoclastic vasculitis, livedo reticularis, and bullous lesions. Skin lesions in SLE occur in sun-exposed areas as a malar rash with photosensitivity in a majority of the cases [6]. The most common form of chronic CLE is DLE, which affects the face and scalp [7]. Genital lupus lesions are a rare manifestation of lupus. This may be due to low prevalence, as well as physician and patient discomfort when trying to locate this manifestation. Unless specifically asked by the doctor, patients rarely disclose genital lesions [8]. The vulvar CD is an uncommon condition characterized by granulomatous vaginal inflammation that does not occur in conjunction with fistulizing CD. The vulvar CD can cause debilitating lymphedema, disfiguring anatomic alterations, secondary abscesses, cellulitis, and squamous cell carcinoma (SCC) if left untreated [9]. Although mucosal involvement in SLE presents as ulcers in the mouth and nose in 95% of cases, a few may present with genital ulcers. Very few studies have reported genital ulcers in SLE patients, and those reported were treated successfully with IV steroids. The following differential diagnosis should be kept in mind when a patient presents with genital ulcers (Table 1).

**Table 1:** Differential diagnosis of genital ulcers STD: sexually transmitted disease

STD-related	1. Syphilis
	2. Herpes genitalis
	3. Chancroid
	4. Granuloma inguinale
	5. Lymphogranuloma venereum
Idiopathic	1. Bechet's disease
	2. Aphthous ulcer
	3. Lipschutz ulcer
Primary malignancy	1. Squamous cell carcinoma
	2. Malignant melanoma
	3. Basal cell carcinoma
Secondary malignancy	1. Leukemia
	2. Choriocarcinoma
Systemic disease-related	1. Lupus erythematosus
	2. Crohn's disease
	3. Lichen planus
	4. Lichen sclerosis
Dermatological disorder	1. Contact dermatitis
	2. Psoriasis
Traumatic ulcer	1. Change into a septic ulcer
	2. Pyogenic ulcer

## Conclusions

Given that SLE genital ulcers are rare entities, genital lupus ulcers are often underdiagnosed. Any patient with a history of SLE presenting with perineal pain has to be evaluated without delay as it causes severe discomfort and declining functionality for the patients. If left untreated, these lesions could progress to SCC. It is always a good practice to perform a thorough physical and genital examination to establish the diagnosis and manage the patients in a timely manner while conducting workups for infective and other inflammatory causes. It is difficult to distinguish lupus genital ulcers from vulvar CD ulcers by physical examination. The only way to confirm and differentiate between genital SLE vs. genital CD is via biopsy and immunofluorescence tests. The genital lesions in SLE present as small papular ulcers with well-demarcated borders and medium-sized erosions that have to be managed surgically. The wedge biopsy results show epidermis with irregular acanthosis with ulceration and dermis infiltrated by dense lymphoplasmacytic infiltrates with fewer neutrophils. It also invades the vessel wall showing fibroid necrosis. After a correct diagnosis of SLE ulcer, our patient responded well to steroids.
